# Unraveling new molecular players involved in the autoregulation of nodulation in *Medicago truncatula*

**DOI:** 10.1093/jxb/ery465

**Published:** 2019-02-08

**Authors:** Pierre Gautrat, Virginie Mortier, Carole Laffont, Annick De Keyser, Justine Fromentin, Florian Frugier, Sofie Goormachtig

**Affiliations:** 1 Institute of Plant Sciences-Paris Saclay (IPS2), Centre National de la Recherche Scientifique, Université Paris-Sud, Université Paris-Diderot, Université d’Evry, Institut National de la Recherche Agronomique, Université Paris-Saclay, Gif-sur-Yvette, France; 2 Department of Plant Biotechnology and Bioinformatics, Ghent University, Ghent, Belgium; 3 Center for Plant Systems Biology, VIB, Ghent, Belgium; 4 Laboratoire des Interactions Plantes-Microorganismes, Unité Mixte de Recherche, Institut National de la Recherche Agronomique, Castanet-Tolosan, France; 5 Laboratoire des Interactions Plantes-Microorganismes, Unité Mixte de Recherche, Centre National de la Recherche Scientifique, Castanet-Tolosan, France

**Keywords:** Autoregulation of nodulation (AON), CLAVATA signaling peptide, F-box protein, Nod factor perception (NFP), rhizobia, symbiotic nodulation

## Abstract

The number of legume root nodules resulting from a symbiosis with rhizobia is tightly controlled by the plant. Certain members of the CLAVATA3/Embryo Surrounding Region (CLE) peptide family, specifically MtCLE12 and MtCLE13 in *Medicago truncatula*, act in the systemic autoregulation of nodulation (AON) pathway that negatively regulates the number of nodules. Little is known about the molecular pathways that operate downstream of the AON-related CLE peptides. Here, by means of a transcriptome analysis, we show that roots ectopically expressing *MtCLE13* deregulate only a limited number of genes, including three down-regulated genes encoding lysin motif receptor-like kinases (LysM-RLKs), among which are the nodulation factor (NF) receptor *NF Perception* gene (*NFP*) and two up-regulated genes, *MtTML1* and *MtTML2,* encoding Too Much Love (TML)-related Kelch-repeat containing F-box proteins. The observed deregulation was specific for the ectopic expression of nodulation-related *MtCLE* genes and depended on the Super Numeric Nodules (SUNN) AON RLK. Moreover, overexpression and silencing of these two *MtTML* genes demonstrated that they play a role in the negative regulation of nodule numbers. Hence, the identified *MtTML* genes are the functional counterpart of the *Lotus japonicus TML* gene shown to be central in the AON pathway. Additionally, we propose that the down-regulation of a subset of LysM-RLK-encoding genes, among which is *NFP*, might contribute to the restriction of further nodulation once the first nodules have been formed.

## Teaser

We provide new insights into the autoregulation of nodulation that allow us to better understand how the legume *Medicago truncatula* restricts further nodulation once enough nodules have been formed.

## Introduction

Under nitrogen-limited conditions, legume plants interact with bacteria, rhizobia, to form symbiotic root nodules, in which the rhizobia fix nitrogen for the benefit of the plant (Oldroyd, 2013; Suzaki *et al.*, 2015; Gamas *et al.*, 2017). Nodulation is initiated through a chemical signal exchange between both symbiotic partners. After the host-derived flavonoids are perceived, the rhizobia secrete lipo-chitooligosaccharides, designated nodulation factors (NFs), which are sensed by the plant root epidermis to activate downstream symbiotic signaling (Fliegmann and Bono, 2015). In the model legume, *Medicago truncatula* (barrel medic), mutation of the lysin motif (LysM) receptor-like kinase (RLK) gene *NF Perception* (*NFP*) abolished nodulation from the earliest initiation stage onward and eliminated root hair curling and activation of (pre-)infection markers, such as *Early NODulin 11* (*ENOD11*) (Ben Amor *et al.*, 2003). Additionally, a related protein, LysM domain RLK 3 (LYK3), has been shown to be involved in NF perception, because its down-regulation by RNAi inhibited early nodulation (Limpens *et al.*, 2003). Also in *Lotus japonicus*, two receptor proteins, NF receptor 5 (LjNFR5) and LjNFR1, that are related to MtNFP and MtLYK3, respectively, control the NF signaling (Madsen *et al.*, 2003; Radutoiu *et al.*, 2003). LYK3 and NFR1 are functional kinases that can form a complex with the NFR5/NFP non-functional kinases (Fliegmann and Bono, 2015). Moreover, knock-down of *MtLYK4* also affects rhizobial infections, hinting at a functional redundancy between MtLYK3 and MtLYK4 for nodulation initiation (Limpens *et al.*, 2003). In *L. japonicus*, direct binding of NFs on an NFR5/NFR1 heterodimeric complex was reported. An additional LysM-RLK gene, the high-affinity lipo-chitooligosaccharide-binding protein 3 gene (*LYR3*), was detected as a high-affinity NF-binding protein that interacts with MtLYK3, although the *lyr3* mutants did not reveal any nodulation phenotype (Fliegmann *et al.*, 2013, 2016; Fliegmann and Bono, 2015). Other LysM-RLKs have been connected with early nodulation: the ExoPolysaccharide Receptor 3 (LjEPR3) that is linked to the perception of another type of rhizobial signals, the exopolysaccharides, and that acts downstream of NFR1/NFR5 (Kawaharada *et al.*, 2017) and the epidermal LysM receptor (NFRe) that has been proposed to maintain the response of epidermal cells to rhizobia along the expanding root system (Murakami *et al.*, 2018). In *M. truncatula* as well as in other legumes, several LysM-RLK-encoding genes exist, the potential functions of which in relation to symbiotic nodulation remain to be established.

Nitrogen-fixing symbiotic nodulation is an adaptation of legume plants to nitrogen-starved conditions and is, therefore, tightly controlled by the host plant. One of the negative regulatory pathways that limits the nodule formation dependent on the metabolic status of the shoot (carbon) and root (nitrogen) is the long-distance (systemic) autoregulation of nodulation (AON) pathway (Suzaki *et al.*, 2015). Early after rhizobial infection, the transcription of a subset of genes encoding CLAVATA3/Embryo Surrounding Region (CLE) peptides, the so-called MtCLE12 and MtCLE13 in *M. truncatula*, is triggered to activate the AON (Mortier *et al.*, 2010). Similarly, in *L. japonicus*, the LjCLE-root signaling (RS)1, LjCLE-RS2, and LjCLE-RS3 peptide-encoding genes are induced by rhizobia and/or nitrogen (Okamoto *et al.*, 2009; Nishida *et al.*, 2016). Interestingly, these genes are orthologous to *MtCLE12* and *MtCLE13*, as well as to the rhizobia-induced CLE 1 (RIC1) and RIC2 peptide-encoding genes in *Glycine max* (soybean) and *Phaseolus vulgaris* (common bean) (Reid *et al.*, 2011; Lim *et al.*, 2011; Hastwell *et al.*, 2017). CLE peptides are 12–13 amino acids long and are secreted as signaling peptides derived from the C-terminal region of pre-proproteins that have been shown to act as non-cell-autonomous signals in various developmental contexts (Araya *et al.*, 2016; Yamaguchi *et al.*, 2016). In *Arabidopsis thaliana* (thale cress), their function is mostly associated with the regulation of cell proliferation and differentiation during plant development, notably in the shoot and root apical meristems and in the cambium meristem in relation to tracheary element differentiation. The MtCLE12, but not MtCLE13, peptides are tri-arabinosylated, possibly by an enzyme from the hydroxyproline *O*-arabinosyltransferase (HPAT) family encoded by the *M. truncatula Root Determined Nodulation 1* (*RDN1*) gene, mutation of which leads to excessive nodulation (Schnabel *et al.*, 2011; Kassaw *et al.*, 2017). The negative effect of AON-related CLE peptides on the nodule number relies on a shoot-acting leucine-rich repeat (LRR) RLK, designated Super Numeric Nodules (SUNN) in *M. truncatula*, Hypernodulation Aberrant Root Formation (HAR1) in *L. japonicus*, Nodule Autoregulation Receptor Kinase (NARK) in soybean, and Symbiosis29 (SYM29) in *Pisum sativum* (pea) (Krusell *et al.*, 2002; Searle *et al.*, 2003; Schnabel *et al.*, 2005; Suzaki *et al.*, 2015). A transcript profiling analysis of inoculated and uninoculated *nark* soybean leaves revealed a differential expression of the jasmonic acid biosynthesis and response genes (Kinkema and Gresshoff, 2008), suggesting that a shoot-specific down-regulation of the jasmonic acid response genes by rhizobial inoculation might mediate the AON, at least in soybean. The AON may involve a SUNN-dependent regulation of the long-distance shoot-to-root polar auxin transport in *M. trunculata* (van Noorden *et al.*, 2006) and the down-regulation of a specific mobile miRNA in *L. japonicus* (Tsikou *et al.*, 2018).

Regarding the downstream targets of the MtCLE12/MtCLE13–MtSUNN pathway in roots, only very few fragmentary data are currently available (Schnabel *et al.*, 2010; Suzaki *et al.*, 2015). Therefore, there is a real need to expand knowledge of the downstream effectors of the AON pathway. In *M. truncatula*, the systemic AON pathway has been shown possibly to inhibit the NF signaling pathway, because the early nodulation marker *MtENOD11* was no longer activated in roots ectopically expressing *MtCLE13* after rhizobial inoculation (Mortier *et al.*, 2010). In contrast, in *L. japonicus*, the AON might act on nodule organogenesis (Takahara *et al.*, 2013). Genetic analyses in *L. japonicus* revealed that the *too much love* (*tml*) mutant is affected in a gene acting downstream of the LjCLE–RS/LjHAR1 pathway in roots (Magori *et al.*, 2009; Takahara *et al.*, 2013). The *LjTML* gene encodes a Kelch-repeat F-box protein that is probably involved in the targeted ubiquitin-dependent proteolysis of still unknown nuclear proteins that are expected to be critical for early nodulation. Recently, the *TML* transcript level has been shown to be controlled in roots by a shoot-derived systemic miRNA, the miR2111, the expression of which is up-regulated during nodulation in an LjHAR1-dependent manner (Tsikou *et al.*, 2018), but whether the *CLE RS1/RS2* expression affects the *TML* expression is still unknown.

To investigate the downstream molecular pathways activated by AON-related CLE peptides in *M. truncatula*, we analyzed the transcriptome of roots ectopically expressing *MtCLE13*. *MtCLE13* was selected because its induction during nodulation occurs earlier than that of *MtCLE12* (Mortier *et al.*, 2010). We show that only a limited set of root genes were differentially regulated, including *NFP* and some *LysM-RLK*-related genes, as well as two *TML* orthologs. Together, these results suggest that in *M. truncatula* AON-related CLE peptides act through the root activity of TML F-box proteins and could inhibit nodule formation via the down-regulation of genes involved in NF perception, such as *NFP* and other related *LysM-RLK* genes.

## Materials and methods

### Biological material

*Medicago truncatula* Gaertn. cv Jemalong A17, the *pNFP:GUS* stable transgenic line (Arrighi *et al.*, 2006), and the *sunn-4* mutant (Sagan *et al.*, 1995; Schnabel *et al.*, 2005) were grown and inoculated as described (Mergaert *et al.*, 2003). The *Sinorhizobium meliloti* Sm1021 strain and the *Agrobacterium rhizogenes* Arqua1 strain were grown at 28 °C in a yeast extract broth (YEB) medium supplemented with 50 mg l^–1^ streptomycin.

For the quantitative reverse transcription–PCR (qRT–PCR) analysis, plants were grown *in vitro* in square Petri dishes (12×12 cm) on a low nitrogen ‘i’ agar medium (0.125 mM KNO_3_; Blondon, 1964). For the nodulation kinetics, nodules were harvested 1–15 d after inoculation with *S. meliloti* from plants grown *in vitro* on nitrogen-poor ‘i’ medium. The symbiotic rhizobia-responsive zone, located above the root tip, was isolated from uninoculated roots and used as control. For the *Agrobacterium rhizogenes*-mediated transgenic root transformation, nitrogen-deprived Fahraeus medium was used (0.01 mM KNO_3_; Truchet *et al.*, 1989).

### Cloning procedures

The *35S:MtCLE4*, *35S:MtCLE12*, and *35S:MtCLE13* vectors were generated as described (Mortier *et al.*, 2010). The ORFs of *MtTML1* and *MtTML2* were amplified from *M. truncatula* genomic DNA. Primers containing the attB sequences at their 5' end were used for amplification of Gateway cloning ready products (see Supplementary Table S1 at *JXB* online) into the pDONR207 vector by means of the Gateway BP recombinase (Invitrogen, Carlsbad, CA, USA). After verification by sequencing, constructs were transferred via an LR recombinase reaction into the pK7WG2D binary vector (Karimi *et al.*, 2002), allowing the expression of the *MtTML1* or *MtTML2* genes under the control of the Cauliflower mosaic virus 35S promoter.

RNAi constructs were designed to target both the *MtTML1* and *MtTML2* genes within the region that is the most conserved at the nucleotide level between the two genes. The *MtTML1* and *MtTML2* RNAi constructs were amplified with primers specific for the *MtTML1* or *MtTML2* genes, respectively (Supplementary Table S1). These PCR products were cloned with the Gateway technology in the pENTR/D-TOPO vector (Thermo Scientific, Waltham, MA, USA) and then in the pFRN destination vector (Gonzalez-Rizzo *et al.*, 2006). The final RNAi constructs were sequenced for validation. The β-glucuronidase (GUS) RNAi control vector has been published previously (Gonzalez-Rizzo *et al*., 2006).

### RNA extraction, cDNA synthesis, and qRT–PCR analysis

Total RNA was isolated with the RNeasy Plant mini kit (Qiagen, Hilden, Germany) according to the manufacturer’s instructions. After a DNase treatment, samples were purified by NH_4_Ac (5 M) precipitation, quality controlled, and quantified with a Nanodrop spectrophotometer (Isogen, Hackensack, NJ, USA). RNA (2 μg) was used for cDNA synthesis with the Superscript Reverse Transcriptase Kit (Invitrogen). The qRT–PCR experiments were done on a LightCycler 480 (Roche Diagnostics, Brussels, Belgium), and SYBR Green was used for detection. Cycle threshold values were obtained and analyzed by the 2^-ΔΔC^T method (Livak and Schmittgen, 2001). The values from at least three biological replicates and three technical replicates were normalized against genes encoding histone 3-like, actin 11, or ubiquitin, that had been selected as reference genes with the Genorm software (https://genorm.cmgg.be/). Values were calibrated with the control sample (control vector, untreated condition) to highlight fold changes. All primers used are indicated in Supplementary Table S1.

### *Agrobacterium rhizogenes*-mediated transgenic root transformation

The protocol used has been described previously (Boisson-Dernier *et al.*, 2001). Transgenic roots overexpressing either the *MtCLE* or *MtTML* genes were selected based on a green fluorescent protein marker present in the binary vector with an MZFLII stereomicroscope (Leica Microsystems,, Wetzlar, Germany) equipped with a blue light source and a Leica green fluorescent protein plus filter set. Transgenic roots expressing RNAi constructs were selected on 25 mg l^–1^ kanamycin (Sigma-Aldrich, St. Louis, MO, USA).

### Transcriptomic analysis

Roots expressing either the *35S:GUS* or the *35S:MtCLE13* constructs were obtained by means of an *A. rhizogenes* transformation, as described previously (Mortier *et al.*, 2010), and grown in an aeroponic system on a nitrogen minimal medium ‘i’ (Blondon, 1964). At 40 days post-germination, transgenic roots of ~15 plants were harvested and pooled for RNA isolation as described (Mortier *et al.*, 2010). Samples were hybridized to the Affymetrix Gene Chip *M. truncatula* genome array (http://www.affymetrix.com). RNA processing, probe hybridization, washing, and scanning of the arrays were carried out at the VIB MicroArray Facility (Leuven, Belgium).

### Histochemical localization of GUS activity

The GUS activity of *pNFP:GUS* roots was analyzed with 5-bromo-4-chloro-3-indolyl-β-d-glucuronic acid as substrate (Sigma-Aldrich) (Mortier *et al.*, 2010). Roots and nodule primordia were vacuum infiltrated for 20 min and subsequently incubated at 37 °C for 4 h. After staining, roots and root nodule primordia were fixed and dehydrated in 70% ethanol as described (Mortier *et al.*, 2010). Photographs were taken with an MZFLII stereomicroscope (Leica Microsystems).

### Protein similarity tree analysis

To identify MtTML1-related proteins, the full amino acid sequence of MtTML1 was analyzed with the BLASTP algorithm on the NCBI database (https://www.ncbi.nlm.nih.gov/BLAST/) for *M. truncatula* and *A. thaliana*, and with the Lotus Genome Browser (https://lotus.au.dk/genome/) for *L. japonicus*. The sequences from the three proteins most closely related to MtTML1 were retrieved for each species, and the F-box protein Transport Inhibitor Response 1 (AtTIR1) from *A. thaliana* (At3g62980, TAIR database; https://www.arabidopsis.org/) was used to anchor the tree. For the generation of a similarity tree by means of the SeaView version 4 program (Gouy *et al.*, 2010), the complete amino acid sequence was utilized for multiple alignments with MUSCLE (Edgar, 2004). Poorly aligned positions and divergent regions were eliminated with the G-blocks software (Castresana, 2000). A maximum-likelihood phylogenetic tree was obtained with the PhyML program (Guindon *et al.*, 2010) as well as an approximate likelihood-ratio test for branch support.

### Statistical analyses

For the microarray analysis, data were preprocessed with the ‘Robust Multiarray Averaging’ (RMA) algorithm (Irizarry *et al.*, 2003) that involves three steps: background adjustment of RMA convolution, quantile normalization, and summarization with the median polish algorithm—in which the median values per probe set, adjusted for slide differences, are calculated. Based on an empirical Bayes moderated *t*-test (Smyth, 2004), as implemented in the Bioconductor package Linear Models for Microarray Data (LIMMA) (Ritchie *et al.*, 2015), *P*-values were calculated to measure the differential expression between *35:GUS* (control) and *35S:MtCLE13* transgenic roots. The *P*-values were then corrected for multiple testing problems to control the false discovery rate (FDR) (Benjamini and Hochberg, 1995). Threshold values for differential expression fold changes were set at 1.5 and at 0.05 for adjusted *P*-values (FDR).

For the frequency of GUS-positive roots, three biological repeats were analyzed and significant differences were identified with a χ^2^ test (*P*<0.01). For the nodulation phenotypes, a Kruskal–Wallis test was done (α<0.01) with the Xlstat software (https://www.xlstat.com/fr/). For qRT–PCR analyses, normalized values were examined with an ANOVA mixed model procedure with the SAS Enterprise 5.1 software (https://support.sas.com/en/support-home.html).

## Results

### Identification of downstream effectors of CLE peptide signaling during nodulation by means of a transcriptome analysis

Previously, *MtCLE13* overexpression in *M. truncatula* roots had been shown to inhibit nodulation before the induction of *MtENOD11* expression by *S. meliloti* (Mortier *et al.*, 2010). We hypothesized that the inhibitory factor(s) could already be present in roots overexpressing the *MtCLE13* gene prior to inoculation. Hence, the transcriptome of non-inoculated roots expressing either a *35S:GUS* (control) or a *35S:MtCLE13* construct was compared. This analysis revealed that only a limited number of genes were differentially expressed. Considering a stringent threshold for the adjusted *P-*values (|FDR|<0.05), 17 differentially expressed Affymetrix microarray probes (corresponding to 16 genes) were identified between the control and *35S:MtCLE13* roots (Supplementary Dataset S1).

**Fig. 1.**
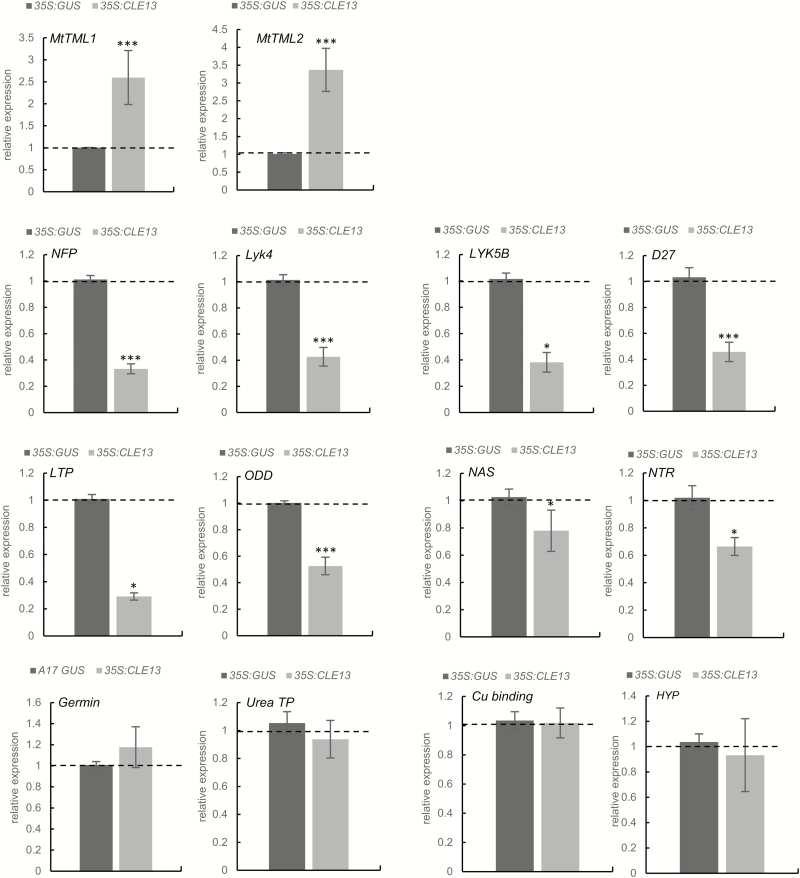
qRT–PCR validation of genes differentially expressed in *35S:MtCLE13* roots. Expression analysis by qRT–PCR identified different genes as deregulated in *35S:MtCLE13* roots compared with *35S:GUS* (control) roots with the microarray approach (see Suppementary Dataset S1). Gene expression was calibrated relative to the *35S:GUS* control roots to highlight fold changes, as indicated by the dotted line. Error bars represent the SE of the mean of at least three biological repeats (*n*>15 plants per biological replicate). *P<0.05; **P<0.01; ***P<0.001 indicate significant differences between the two genotypes as measured with an ANOVA mixed model with a Tukey’s post-hoc comparison. The gene expression levels were calibrated against the expression found in the *35S:GUS* control roots. *TML1* and *TML2*, Too Much Love 1 and *2*; *NFP*, Nod Factor perception; *LYK4*, LysM receptor-like kinase (RLK) 4; *LYK5B*, LysM-RLK 5B; *D27*, β-carotene isomerase; *LTP*, lipid transfer protein; *ODD*, oxoglutarate-dependent dioxygenase; *NAS*, nicotianamide synthase; *NTR*, MtNRT2.3 high-affinity nitrate transporter; Urea TP, urea transporter; Cu-binding, copper-binding protein; HyP, hypothetical protein.

The most differentially expressed gene corresponded to *MtCLE13*, validating the efficiency of the overexpression strategy (Supplementary Dataset S1). Additionally, an up-regulation of two Kelch repeat-containing F-box protein-encoding genes in *35S:MtCLE13* roots was detected. Initially, three hits were discovered, but further sequence analyses uncovered that two Affymetrix probes (Mtr13058.1.S1_at and Mtr. 9802.1.S1_at) corresponded to the same gene. As the closest homolog in *L. japonicus* for these two genes was *LjTML*, we designated these genes *MtTML1* (Medtr7g029290; MtGI v4.0) and *MtTML2* (Medtr6g023805). Comparison of the proteins revealed that LjTML was 89% and 73% similar to MtTML1 and MtTML2, respectively. A similarity tree was generated with the three proteins from *M. truncatula*, *L. japonicus*, and *A. thaliana* most closely related to MtTML1 (Supplementary Fig S1). The analysis revealed that there was no equivalent duplication in *L. japonicus* compared with that identified for *M. truncatula*, suggesting that the nodulation-related function of TML proteins does not correlate with a specific duplication pattern. A qRT–PCR analysis validated the increased expression of *TML1* and *TML2* in independent *35S:CLE13* root samples (Fig. 1). Among the genes identified as down-regulated in the transcriptomic analysis of the *35S:MtCLE13* roots and validated by qRT–PCR on independent samples (Fig. 1), a striking enrichment for LysM-RLK-encoding genes was found, including *NFP*, *LYK4*, and a gene coding for a truncated LysM-RLK protein most closely related to LYK5 without a kinase domain, the so-called ‘LYK5B’ (Fig. 1) (Larrainzar *et al.*, 2015; Bono and Cullimore, 2019).

In addition, genes coding for several enzymes were detected as down-regulated in both microarrays and the qRT–PCR analysis: a D27 β-carotene isomerase, a lipid transfer protein (LTP), a 2-oxoglutarate-dependent dioxygenase (ODD), and a nicotianamine synthase (NAS). Finally, the microarray analysis identified up-regulated genes in *35S:MtCLE13* roots encoding a high-affinity nitrate transporter (MtNRT2.3) (Pellizzaro *et al.*, 2015), a sodium:solute symporter/urea-proton symporter family protein (Kojima *et al.*, 2007), a copper-binding protein, a germin-like protein, and two hypothetical proteins. However, independent qRT–PCR validations could not confirm these expression patterns (Fig. 1) and no expression could be detected for one of the genes encoding a hypothetical protein. Overall, this transcriptomic analysis highlighted a limited number of genes, of which the expression is affected by the ectopic expression of *MtCLE13* in roots, including genes encoding a subset of LysM-RLK proteins and TML-like proteins linked to the symbiotic NF perception and to the *L. japonicus* AON pathway, respectively.

### *MtCLE12* and *MtCLE13* regulate the expression of *NFP* and *TML* in a SUNN-dependent manner

**Fig. 2.**
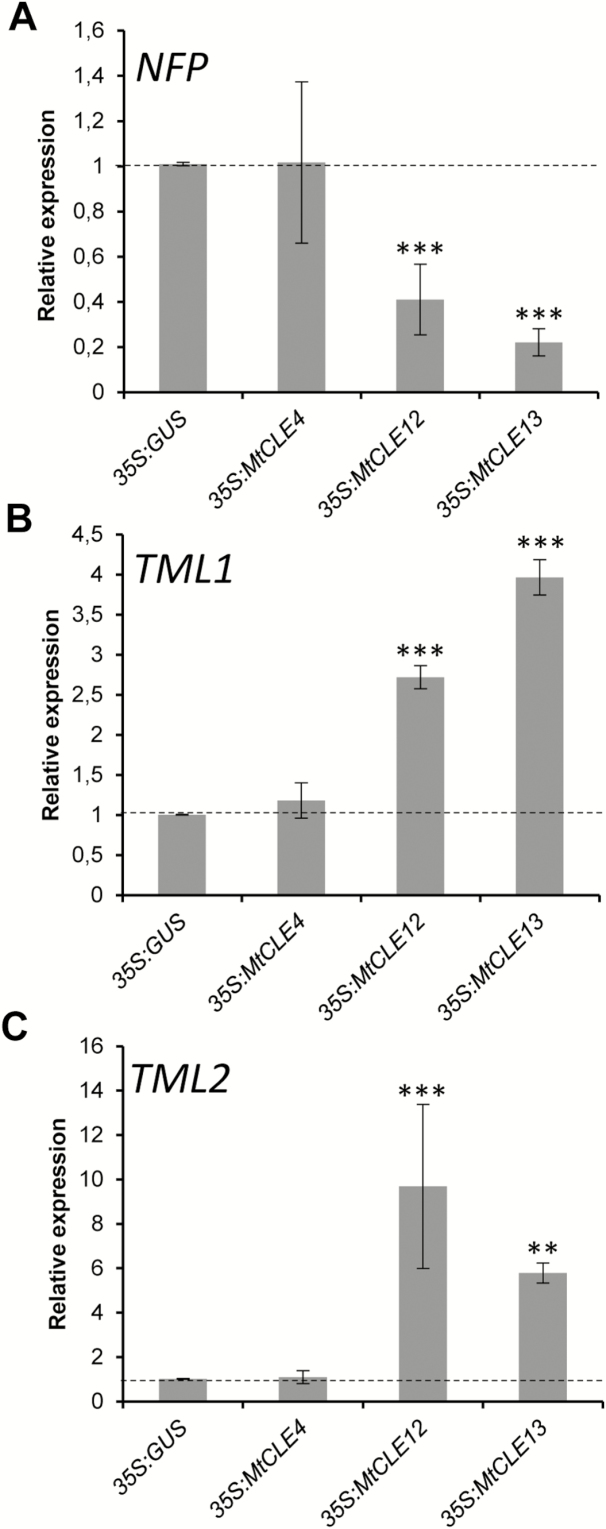
Expression of the *M. truncatula NFP*, *TML1*, and *TML2* genes in CLE peptide-overexpressing roots. (A–C) Expression analysis by qRT–CR of *NFP* (A), *TML1* (B), and *TML2* (C) in roots expressing a *35S:GUS* (control), a *35S:MtCLE4* (a non-AON-related CLE control), a *35S:MtCLE12*, or a *35S:MtCLE13* construct. The gene expression levels are shown relative to the expression found in the *35S:GUS* control roots. To highlight the fold changes, these control levels are indicated by the dotted line. Error bars represent the SE of the mean of three biological repeats (*n*>15 plants per biological replicate). *P<0.05; **P<0.01; ***P<0.001, significant differences from the levels observed in the *35S:GUS* genotype as found with an ANOVA mixed model with a Tukey’s post-hoc comparison.

To determine whether the regulation of the previously nodulation-linked *NFP* and *TML1/TML2* genes was specific for the ectopic *MtCLE13* expression, we analyzed their expression in roots expressing *MtCLE12* or the non-AON-related *MtCLE4* gene (Mortier *et al.*, 2010). First, the expression of the *GUS*, *MtCLE4*, *MtCLE12*, or *MtCLE13* transgenes was controlled specifically in the corresponding overexpressing roots, validating the sampling procedure (Supplementary Fig S2). Interestingly, the ectopic expression of *MtCLE12*, like that of *MtCLE13*, also reduced *NFP* expression (Fig. 2A) and increased *TML1* and *TML2* expression (Fig. 2B, C). Importantly, the expression of *NFP* or *TML1/TML2* was not altered by the ectopic *MtCLE4* expression (Fig. 2).

**Fig. 3.**
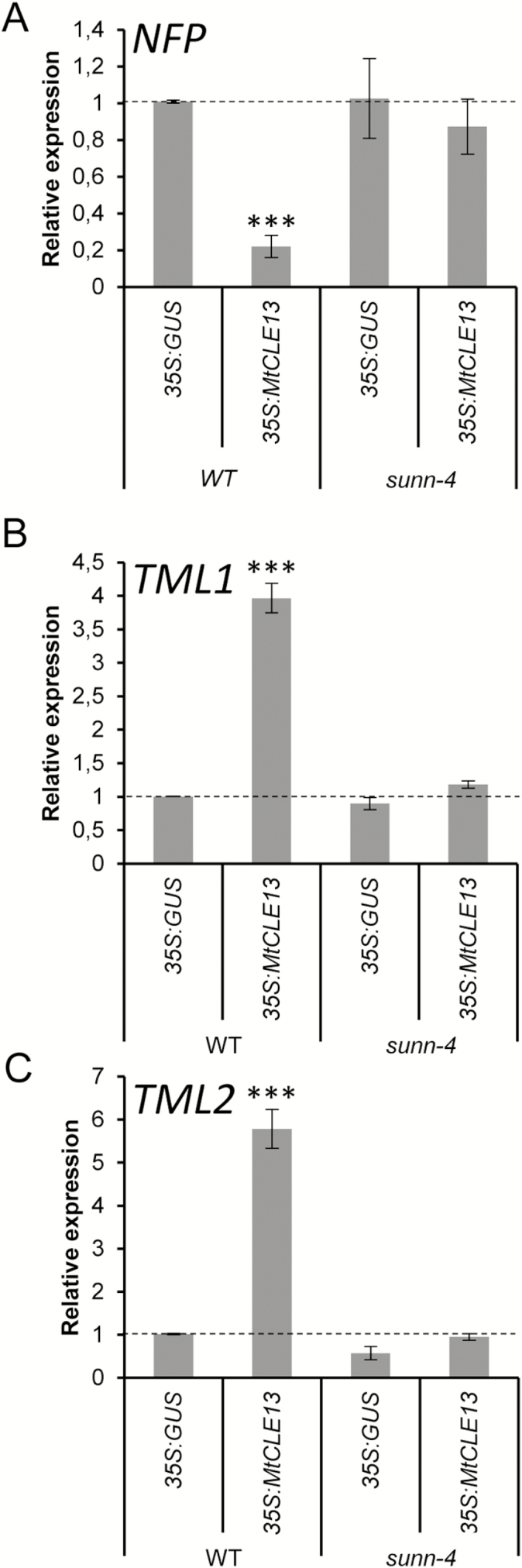
Expression of the *M. truncatula NFP*, *TML1*, and *TML2* genes in the *sunn* mutant. (A–C) Expression analysis by qRT–PCR of *NFP* (A), *TML1* (B), and *TML2* (C) in wild-type (WT) or *sunn-4* mutant roots, expressing a *35S:GUS* (control) or a *35S:MtCLE13* construct. Gene expression is shown relative to the expression found in the *35S:GUS* control roots and highlighted by the dotted line. Error bars represent the SE of the mean of three biological repeats (*n*>15 plants per replicate). The data of the WT plants are the same as those shown in Fig. 2. *P<0.05; **P<0.01; ***P<0.001, significant differences from the levels observed in the *35S:GUS* genotype as found with an ANOVA mixed model with a Tukey’s post-hoc comparison.

As the nodulation inhibition provoked by the ectopic *MtCLE13* expression depends on SUNN within the systemic AON pathway (Mortier *et al.*, 2012), we wanted to determine whether the *MtCLE13* regulation of *NFP* and *TML1/TML2* expression was also SUNN dependent. The *35S:MtCLE13* or *35S:GUS* constructs were introduced into wild-type and *sunn-4* mutant roots, revealing that the down-regulation of *NFP* and the up-regulation of *TML1/TML2* by the ectopic expression of *MtCLE13* no longer occurred in *sunn-4* mutants (Fig. 3). These data indicate that the *MtCLE13*-mediated regulation of *NFP* and *TML1/TML2* expression relies on MtSUNN and support the hypothesis that down-regulation of *NFP* and up-regulation of *TML1/TML2* are part of the AON mechanism.

### The *NFP* promoter activity is down-regulated in *35S:MtCLE13* roots

**Fig. 4.**
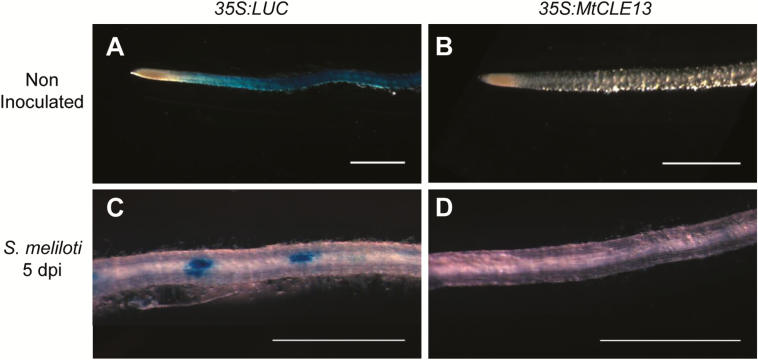
Down-regulated expression of a p*NFP:GUS* transcriptional fusion in roots overexpressing *MtCLE13*. Roots were stained with GUS before (A and B) and after (C and D) inoculation with *S. meliloti* (5 dpi). Representative roots shown express a *35S:LUC* control vector (A and C) or a *35S:MtCLE13* construct (B and D) (*n*>44 independent roots analyzed for each construct and condition, from three different biological experiments). Scale bars=1 mm.

For independent corroboration of the transcriptomic and qRT–PCR datasets, roots of stable *pNFP:GUS* plants were transformed with the *35S:MtCLE13* construct. As a control, *pNFP:GUS* plants were generated with a *35S:LUCIFERASE* (*35S:LUC*) construct. In 50% of the uninoculated control roots, a GUS activity was observed, whereas it occurred in only 18% of the *35S:MtCLE13* roots (Fig. 4A, B). Similarly, at an early stage after *S. meliloti* inoculation (5 dpi), the *pNFP:GUS* activity was observed in 61% of the *35S:LUC* roots, but in only 4% of the *35S:MtCLE13* roots (Fig. 4C, D). Overall, these results support that the ectopic *MtCLE13* expression leads to a down-regulated *NFP* expression already under non-inoculated conditions.

### TML1 and TML2 negatively affect nodule number

**Fig. 5.**
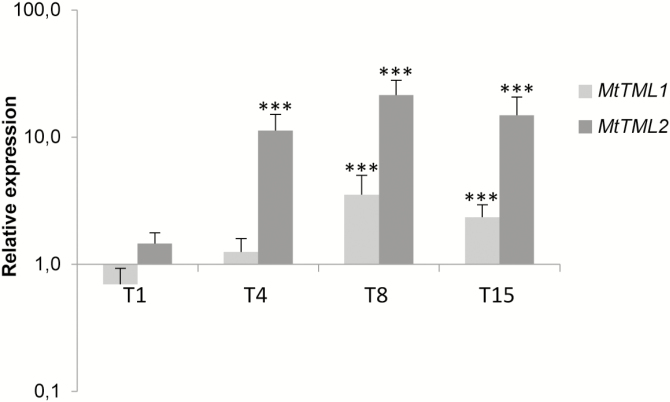
Expression analysis of the two *M. truncatula* genes, *MtTML1* and *MtTML2*, during nodulation. Expression analysis by qRT–PCR of *MtTML1* and *MtTML2* at 1, 4, 8, and 15 dpi with *S. meliloti*. Gene expression was calibrated relative to non-inoculated control root regions susceptible to rhizobia to highlight fold changes. Error bars represent the SD of two biological repeats (*n*>20 plants per biological replicate). *P<0.05; **P<0.01; ***P<0.001, significant differences from the levels observed in the control sample as found with an ANOVA mixed model with a Tukey’s post-hoc comparison.

To determine whether the *TML1* and *TML2* genes could act during nodule development, we analyzed their expression by comparing the rhizobia-susceptible root regions before inoculation and at 1, 4, 8, and 15 dpi. The expression level of *TML1* slightly increased at 4 dpi, but only significantly from 8 dpi onward (Fig. 5). At 4 dpi, the *TML2* transcript level was already clearly higher than that in control roots and than that of *TML1* (Fig. 5), hinting at a function for *TML1* and *TML2* in nodulation.

**Fig. 6.**
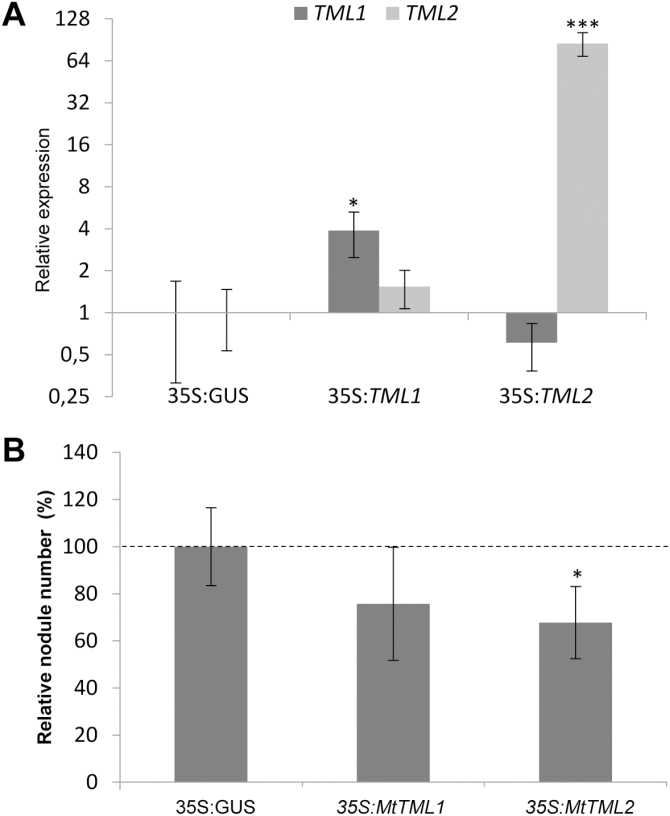
Reduced nodule formation of roots ectopically expressing *MtTML1* and *MtTML2.* (A) Expression analysis of the *MtTML1* and *MtTML2* genes in *35S:MtTML1* and *35S:MtTML2* roots. Gene expression is shown relative to levels found in *35S:GUS* control roots to highlight fold changes. Error bars represent the SD (*n*=6 per biological replicate). *P<0.05; **P<0.01; ***P<0.001, significant differences from the levels observed in the *35S:GUS* genotype as found with an ANOVA mixed model with a post-hoc Tukey comparison. (B) Nodule number per plant in *35S:GUS* (control), *35S:MtTML1*, or *35S:MtTML2* roots at 14 dpi with *S. meliloti*. Data from two independent biological experiments were normalized, relative to the control. Error bars represent confidence intervals (α=0.05; *n*>16 per biological replicate). Significant statistical differences are indicated by asterisks based on a Kruskal–Wallis test (α<0.05).

**Fig. 7.**
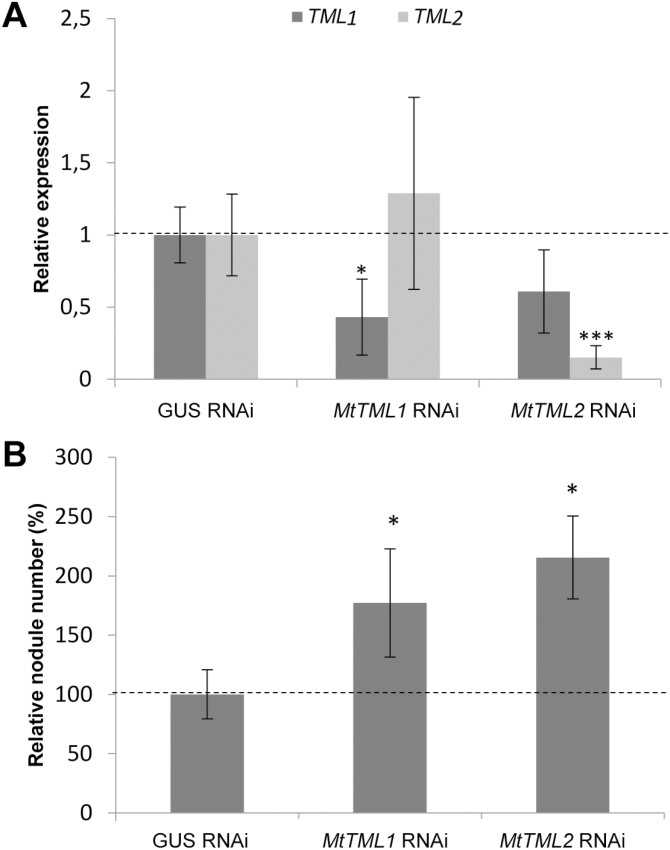
Increased nodule number in *M. truncatula* roots silenced for *MtTML1* and *MtTML2*. (A) Expression analysis of *MtTML1* and *MtTML2* genes in *MtTML1* and *MtTML2* RNAi roots. Gene expression was calibrated relative to *GUS* RNAi control roots to highlight fold changes, as indicated by the dotted line. Error bars represent the SD (*n*=4 per biological replicate). *P<0.05; **P<0.01; ***P<0.001, significant differences from the levels observed in the *35S:GUS* RNAi genotype as found with an ANOVA mixed model with a post-hoc Tukey comparison. (B) Nodule number per plant in *GUS* RNAi (control), *MtTML1* RNAi, or *MtTML2* RNAi roots at 14 dpi with *S. meliloti*. Data from two independent biological experiments were normalized relative to the control. Error bars represent confidence intervals (α=0.05; *n*>13 plants per biological replicate). Significant statistical differences are indicated by asterisks based on a Kruskal–Wallis test (α<0.05).

To evaluate such a potential symbiotic function, we applied an overexpression and an RNAi strategy. The expression of *TML2* was induced at a much higher level in *35S:MtTML2* roots than that of *TML1* in *35S:MtTML1* roots (Fig. 6A). Accordingly, the ectopic expression of *TML2* significantly reduced the number of nodules formed (Fig. 6B). A non-significant reduction was observed in *35S:MtTML1* roots. RNAi constructs targeting preferentially either *TML1* or *TML2* were designed and validated (Fig. 7A), both leading to a significantly increased nodulation efficiency (Fig. 7B). Altogether, these observations indicate that TML1 and TML2 act in roots to negatively control the nodule number.

## Discussion

The two structurally and phylogenically related CLE peptides MtCLE12 and MtCLE13 that are up-regulated during early *M. truncatula* nodulation stages had previously been found to negatively control nodulation based either on ectopic expression or on simultaneous RNAi approaches (Mortier *et al.*, 2010, 2012). A transcriptomic analysis in non-inoculated roots was carried out to analyze the downstream targets of *MtCLE13*, the nodulation-related CLE peptide that is encoded by the gene with the earliest symbiotic expression pattern. The ectopic expression of *MtCLE13* resulted in the down-regulation of the *NFP* gene that encodes a presumptive NF receptor and of two homologous LysM-RLK-encoding genes, *LYK4* and *LYK5B*, the latter of which is a *LYK5* homolog with a truncated kinase domain, indicating that these genes might be putative AON targets. *NFP* mutation and *LYK4* knock-down both reduce nodulation at the initiation stage (Ben Amor *et al.*, 2003; Limpens *et al.*, 2003), clearly implying their symbiotic function, whereas the truncated *LYK5B* had been shown previously to be rapidly controlled by *S. meliloti* inoculation and this in relation to the negative ethylene regulatory pathway (Larrainzar *et al.*, 2015). Accordingly, the use of a *pNFP:GUS* fusion revealed that the ectopic *MtCLE13* expression repressed the *NFP* promoter activity already before *S. meliloti* inoculation in the nodulation-susceptible root zone, possibly contributing to nodulation inhibition. This observation is consistent with the previously reported negative effect of these CLE peptides on nodulation at an early stage, because the *S. meliloti*-induced *ENOD11* expression, an early epidermal infection marker, was reduced in roots ectopically expressing *MtCLE13* (Mortier *et al.*, 2010). In addition, we demonstrated the SUNN-dependent impact of the ectopic *MtCLE13* expression on the *NFP* expression, further supporting the hypothesis that one of the downstream effects of the negative AON pathway might be a reduced *NFP* expression.

Besides *MtCLE13*, another CLE peptide-encoding gene, *MtCLE12*, was linked to the SUNN-dependent AON inhibition of nodulation (Mortier *et al.*, 2010). Accordingly, roots overexpressing *MtCLE12* also led to a down-regulated *NFP* expression. Importantly, ectopic *MtCLE4* expression that does not lead to a decrease in nodule number (Mortier *et al.*, 2010) also does not affect *NFP* expression, hinting at a regulation specificity toward nodulation-related *CLE* genes (Mortier *et al.*, 2010).

The current AON model proposes that once nodulation is initiated, CLE peptides are produced in the roots and are systemically transported to the shoot, where they are perceived by the SUNN receptor, whereafter a feedback signal is delivered to the roots to inhibit nodulation further (Suzaki *et al.*, 2015). Here, we show that the expression of a subset of presumptive NF receptors, including NFP and LYK4, is seemingly targeted by this shoot-to-root signal. Hence, we present a model in which down-regulation of NF perception would inhibit further nodulation events from the initiation stage. Because in *L. japonicus*, nodule primordia and rhizobial infections might both be targets of AON systemic signals (Suzaki *et al.*, 2012; Tsikou *et al.*, 2018), and because depending on the legume species, such as soybean and *Medicago sativa* (alfalfa), an AON effect on infections or on cortical cell divisions had been reported, respectively (Mathews *et al.*, 1989; Caetano-Anollés and Gresshoff, 1991), the AON pathway might inhibit different nodulation steps, even depending on legume species.

In addition to genes encoding LysM-RLK receptors, two genes that code for Kelch repeat-containing F-box proteins most closely related to the *L. japonicus TML* genes were up-regulated by the ectopic expression of MtCLE13, as well as MtCLE12, depending on the SUNN receptor kinase, but not by the non-AON-related MtCLE4 peptide. Accordingly, the expression of both the *MtTML1* and *MtTML2* genes is up-regulated during nodulation. Both gain- and loss-of-function experiments collectively indicated that the *MtTML1* and *MtTML2* genes negatively regulate nodule numbers, as previously reported for *L. japonicus* (Magori *et al.*, 2009). Surprisingly, very different transgene overexpression levels were consistently observed in *35S:MtTML1* or *35S:MtTML2* roots across different independent experiments, possibly hinting at a differential regulation of these two *TML* genes. Such a difference may rely on a post-transcriptional regulation exerted by a miR2111 systemic miRNA, homologous to the one described in *L. japonicus* (Tsikou *et al.*, 2018). Given that the overexpression of AON-related CLE peptides causes *TML* transcript accumulation, it would also be interesting to determine whether the link between the AON-dependent down-regulation of the miR2111 accumulation is associated with known CLE-RS peptides in *L. japonicus*.

Thus, the *M. truncatula* genome contains two closely related genes, *MtTML1* and *MtTML2*, which may be functionally redundant for the negative regulation of nodulation, whereas *L. japonicus* has only one single *TML* gene. This discrepancy might explain why no supernodulating mutant corresponding to a *TML* locus has been identified in *M. truncatula* based on forward genetic screens. Overall, these results suggest that the *TML1* and *TML2* genes are involved in the MtCLE/MtSUNN systemic AON pathway. These genes encode Kelch repeat-containing F-box proteins that are subunits of E3 ubiquitin ligase complexes that specify protein substrates for degradation by the 26S proteasome (Kelley, 2018). A remaining question to be addressed is which nodulation-related proteins interact with and are targeted for degradation by the TML1/TML2 F-box proteins.

Of the 10 other putative target genes of the *MtCLE13* pathway identified, four were confirmed independently by qRT–PCR. One interesting gene that encodes a β-carotene isomerase D27 enzyme involved in strigolactone biosynthesis (Lin *et al.*, 2009) was detected. Strigolactones have previously been linked to symbiotic nodulation in *M. truncatula*, notably based on applications of the synthetic strigolactone *rac*-GR24: low concentrations slightly increased the number of nodules, whereas high concentrations decreased nodulation (De Cuyper *et al.*, 2017). In addition, *MtD27* was rapidly up-regulated by a treatment with NFs in the root epidermis and in nodule primordia (van Zeijl *et al.*, 2015). As the down-regulation of *MtD27* by RNAi did not reveal any nodulation phenotype (van Zeijl *et al.*, 2015), the observed change in *MtD27* expression could be indirectly due to the down-regulation of the *NFP* expression. Hence, a potential link between strigolactones and the AON systemic pathway remains to be established based on alternative approaches. Another gene of which the expression is down-regulated by ectopic *MtCLE13* expression codes for an enzyme from the ODD family that is involved in various aspects of plant metabolism, including the biosynthesis of gibberellins, flavonoids, and flavonoid derivatives (Prescott and John, 1996; Hedden and Phillips, 2000). These different signals are all tightly connected to early nodulation, notably in *M. truncatula*, in which they regulate the production of bacterial NF signals as well as plant NF signaling and nodule organogenesis (Ng *et al.*, 2015; Fonouni-Farde *et al.* 2016*a*, b, 2017; Liu and Murray, 2016). Finally, genes coding for a lipid transfer protein and a nicotianamine synthase were additionally down-regulated by ectopic *MtCLE13* expression. However, speculation about their involvement in the AON pathway is less obvious, and investigation would be needed to examine putative functions.

Altogether, this study provides molecular insights into how the AON–CLE systemic pathway might negatively impact on the nodulation in *M. truncatula* roots. The two functional LjTML homologs identified probably act downstream of the MtSUNN receptor and have a conserved function in the AON, similarly to the *L. japonicus* TML protein (Magori *et al.*, 2009). The detection of the three related LysM-RLK genes, including *NFP* and *LYK4* that are linked to early NF signaling, is very intriguing and suggests that the repression of the NF perception might be one of the early AON targets, explaining the negative regulation of the nodule number.

## Supplementary data

Supplementary data are available at *JXB* online

**Fig. S1.** Protein similarity tree of MtTML1 and MtTML2 with the closely related proteins of *M. truncatula*, *L. japonicus*, and *A. thaliana*.

**Fig. S2.** Expression of *MtCLE* genes in the different *MtCLE-*overexpressing lines.

**Table S1.** List of primers used.

**Dataset S1.** List of genes differentially expressed between *35S:GUS* and *35S:MtCLE13* roots.

Supplement Figures S1-S2

Supplement Dataset S1
