# Supplementary material for: Unraveling new molecular players involved in the autoregulation of nodulation in Medicago truncatula
Source: J Exp Bot. 2019 Feb 8;70(4):1407–17. doi: 10.1093/jxb/ery465 (PMC6382332; doi:10.1093/jxb/ery465)
Supplement: Supplement Figures S1-S2 [file ery465_suppl_supplementary-figures-s1-s2.pdf]

SUPPLEMENTARY INFORMATION

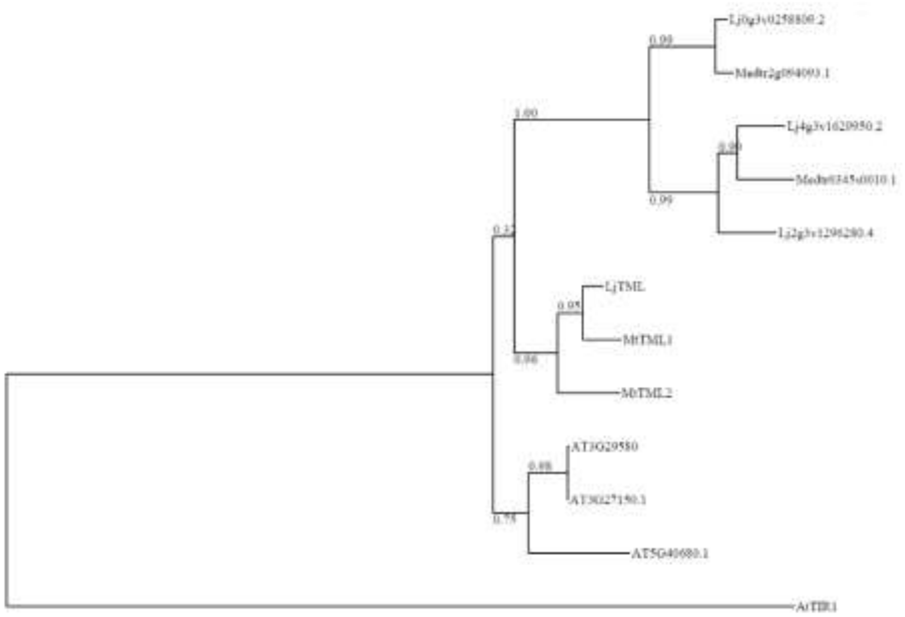

Fig. S1. Protein similarity tree of MtTML1 and MtTML2 with the closely related proteins of *M. truncatula*, *L. japonicus*, and *A. thaliana*.

The tree was designed based on the full amino acid sequences of the three F-box proteins most closely related to TML1 in *M. truncatula*. AtTIR1, an unrelated *A. thaliana* F-box protein was also included to anchor the tree. LRT branch support values are indicated and considered significant when higher than 0.90.

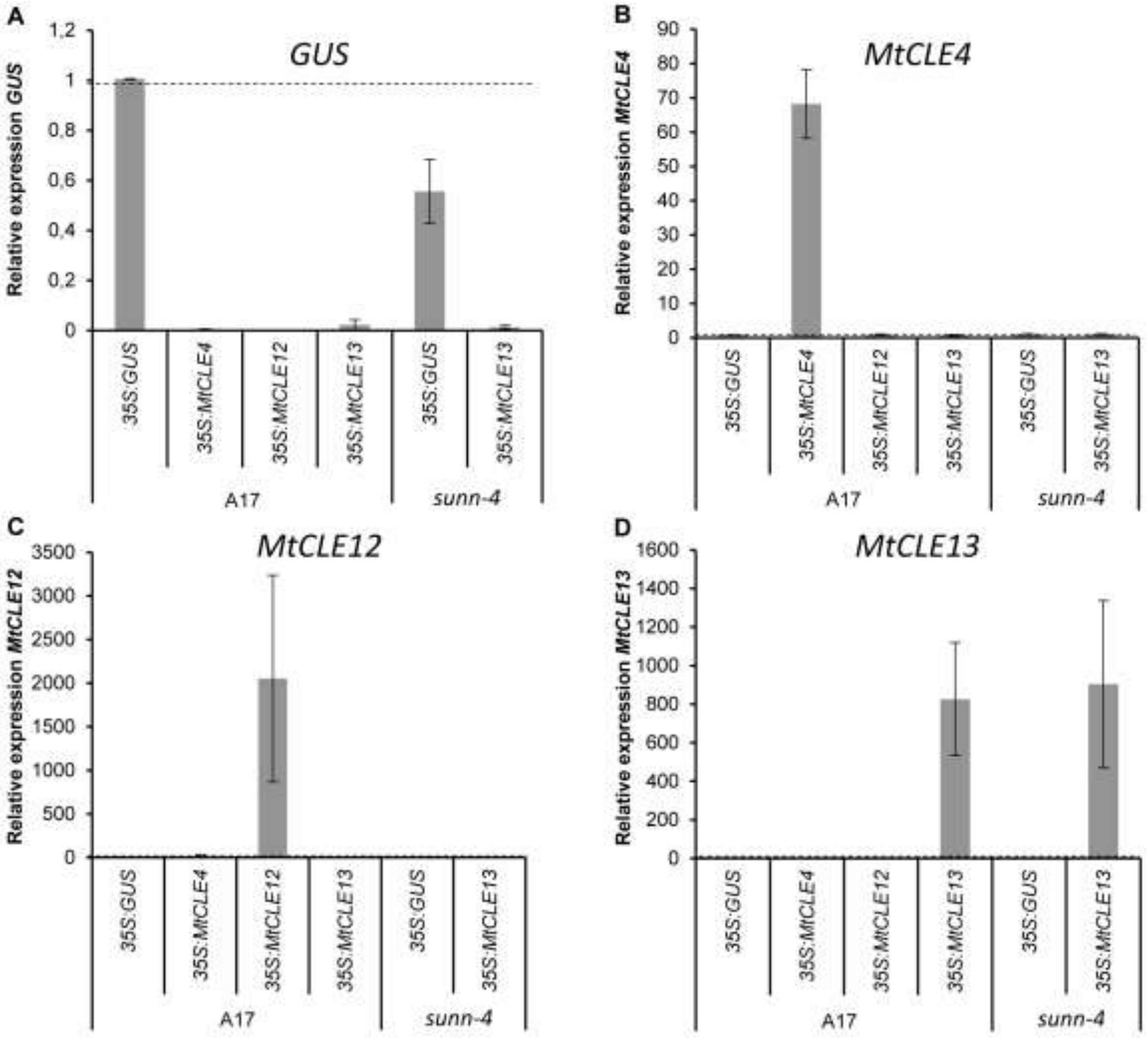

Fig. S2. Expression of *MtCLE* genes in the different *MtCLE*-overexpressing lines. Expression of *GUS* (A), *MtCLE4* (B), *MtCLE12* (C), and *MtCLE13* (D) was measured by qRT-PCR to confirm the specificity of the overexpression constructs in the different lines.

Table S1. List of primers.

Minor cases indicate adapters for Gateway cloning. Y and R in the ubiquitin primers indicate C/T or A/G, respectively.

Gene	*Forward primer*	*Reverse primer*
**qRT-PCR**		
CLE13	CCGAAGCCTTCTACAGAAACTACG	TCTTGGTGGTGATCTTCCATTATGC
Kelch-repeat containing F-box protein (TMLa)	TTTGGCGATTTATACTTGTTGTC	CCATCATCACAGCACAGTTC
Kelch-repeat containing F-box protein (TMLb)	TCTGGTGACAATGGTTCCTC	AAGACATGGTAATGGTAGTAGAAC
NFP	TGGCAGGATAATGACAATGTTAC	GTTGAAGCAGTGAAGTTATGATTG
2-Oxoglutarate-dependent dioxygenase	TCAACCAATTACCGACTCAGG	CATCATCTCTAAACCTTCCACTTG
Urea/proton symporter	TCAGAGTATCAAGGATGGTTGTC	GCATGAAAGCAATAGGAATAACTG
Germin-like	TGTTGCTATTGCTGGACTTAG	ATCAATTATGCTCTTATCCACTTG
Lipid transfer protein	ATTTCAACTCCTGGTCTACTCG	CCGCTCCTCCTCCATCAC
D27	TGCCGTCTTCACCACTATATTC	TCTTCTTCCGTTGATCTCTGAC
Copper binding protein	GAAGCAGAAGGAGAGCCTATAC	CTCAGCACAAGCCTCACAG
Hypothetical protein	TTGCTTGAACCTAACATTTGTATC	TAGTATGTGCAGGAGGAGAAC
Nicotianamine synthase	TCTGTGTGCGTGTGTCTAAG	CAAGGTTTGAGTGTGTCTAAGC
High affinity nitrate transporter	TTGGCTGAATGGAACTCAAAG	TTGTGGTGGTGTAACATCTTC
35S:MtCLE13 transgene	CCTCAGCATAATGGAAGATCAC	AGCGAAACCCTATAAGAACCC
35S:MtCLE4 transgene	ATGCTAACAAGTGAAATGAGCG	CTACCGATTATGCAGAGGATTAGC
35S:MtCLE12 transgene	ATGGAGAATTCAAATAAAGTGCCAAT	TTAGTTATGTATATGGTTTGGTCCAC
GUS	CTACACCACGCCGAACAC	CACCACCTGCCAGTCAAC
Histone 3 Like	ATTCCAAAGGCGGCTGCATA	CTTTGCTTGGTGCTGTTTAGATGG
Ubiquitin	ATGCAGATYTTTGTGAAGAC	ACCACCACGRAGACGGAG
Actin11	TGGCATCACTCAGTACCTTTCAACAG	ACCCAAAGCATCAAATAATAAGTCAACC
**Cloning**		
TMLa - ORF	ggggacaagtttgtacaaaaaagcaggctcaATGACTAATAAAAAAGCATTACC	ggggaccactttgtacaagaaagctgggtaTCAAGTACTTCCCATCATCACAGC
TMLb - ORF	ggggacaagtttgtacaaaaaagcaggctcaATGAGTTATAGTGTGTCTAGTAAAAGATCATTC	ggggaccactttgtacaagaaagctgggtaTCAAGCCAACATCACAGCAC
TMLa RNAi	CACCTGTTGCAGGTGGTTTGGAAA	GGGGAGACTGCGAATCCAAA
TMLb RNAi	CACCTGACGATCTAACATGTGGTGAA	TGCCACACCATAACCAACTTG
